# Dr. J. B. Hamilton

**Published:** 1896-11

**Authors:** 


Dr. J. B. HAMILTON is at present,—or at least was, very recently,—occupying a prominent position in the medical mind of this country, being focused in the perspective on all the journalistic eyes more conspicuously, perhaps, than any other doctor, and by reason, too, it is alleged, of a little personal matter between him and the Surgeon-General of the Marine Hospital Service; a family quarrel.

As far as we can see, most of the journals are in sympathy with the doctor, and make him out a very badly treated man.

The fact is, the doctor is,—has been,—one of the most fortunate, as he is of one of the ablest and most versatile, of our big men. Occupying three conspicuous and remunerative positions, either of which would—should—have satisfied the ambition of any ordinary doctor, as well, perhaps, as filled the measure of his capacity, he doesn't, naturally, want to let go either of them. Being a surgeon in the Marine Hospital Service, he was stationed at Chicago at his request, it seems,—with the promise, on the part of the Surgeon-General, it is claimed—made at a time when they were friends (or, at least, before the difference on the subject of medical legislation by Congress, regarding a public health department, came between them), that he should hold the place two terms. He was in position, therefore, to accept, and did accept, a professorship in the Rush Medical College; a duty that would not take much of his time,—and although his time belonged to the United States, and the Marine Hospital Service had a right to it, yet it is permitted, and all is lovely. Then the editorship of the great A. M. A. *Journal* falls to him with a big salary. Just how much of his time—paid for by the U. S. Treasury Department—was diverted to that work, is not known; but as he has done a great deal of very good work on it, it must have interfered, to some extent, with his duties as a marine hospital surgeon; yet it was permitted, a fact which does not seem to have been duly considered. Surgeon-General Wyman—at the head of the Marine Hospital Service—doubtless, feeling that he had a right to put an officer where he could do most good for the service—ordered Dr. Hamilton to San Francisco—as our readers are already advised.

Hamilton had a good thing, and was vigorously pushing it along. Anything calculated to arrest its progress, would naturally provoke a kick, and Dr. Hamilton kicked; he kicked hard. The Surgeon-General was relentless. The subordinate, — who ought to have known — did know, no doubt, that one of the highest virtues of a soldier is to obey orders, instead of obeying orders, protested; appealed to the Secretary of the Treasury. The Secretary of the Treasury didn't see his way clear, he said, to interfering; so the order was insisted on, and the doctor had to go,—or resign. He resigned. He still has as good a thing as most men of moderate ambition and moderate demands would want.

As we have stated, most of the medical journals have attributed the action of Surgeon-General Wyman to personal resentment, because Dr. Hamilton advocated, through the *Journal* A. M. A., the founding, by Congress, of a separate department of public health, whereas Dr. Wyman's interests, ambition or hopes laid in the direction of a sub-bureau, under control of the Marine Hospital Service, of which he, doubtless. expected to be the head.

Now, we hardly think it fair to thus question the Surgeon-General's motives, though circumstances would seem to warrant a suspicion of the kind. Dr. Wyman is the Supervising

Surgeon-General of the Marine Hospital Service;—a position of great responsibility and trust. He must have the cordial support and co-operation of his officers, or the *morale*, and hence, effectiveness of the service will be crippled. Dr. Hamilton was a valuable officer, an able sanitarian and an experienced quarantine man. He could, in an emergency, be a tower of strength to the general; he was a useful man.

Dr. Wyman must be given credit, at least, for honesty and fidelity in the discharge of his duty. If he needed an able man at the important port on the Pacific coast—he was the best judge of the fitness of his lieutenants for the position, and having selected Dr. Hamilton, he clearly had a right to send him there. So, there are two sides to every question.

If there had been favoritism in putting him at Chicago, as the alleged promise that he should stay there two years, and the remarkable indulgence above mentioned would indicate, it should have been a case of "tickle me and I will tickle you"; and if the Surgeon-General has broken with his alleged favorite, and there was a spice of personality in the matter, Dr. Hamilton has no one to blame but himself; he broke faith first, by turning against what is said to be Dr. Wyman's pet scheme. Wyman is human, if he is great; and tickling, he expected to be tickled in turn. That's politics.

